# Genome-Wide Analysis of RNA Decay in the Cyanobacterium *Synechococcus* sp. Strain PCC 7002

**DOI:** 10.1128/mSystems.00224-20

**Published:** 2020-08-04

**Authors:** Gina C. Gordon, Jeffrey C. Cameron, Sanjan T. P. Gupta, Michael D. Engstrom, Jennifer L. Reed, Brian F. Pfleger

**Affiliations:** a Department of Chemical and Biological Engineering, University of Wisconsin-Madison, Madison, Wisconsin, USA; b Microbiology Doctoral Training Program, University of Wisconsin-Madison, Madison, Wisconsin, USA; University of Tennessee at Knoxville

**Keywords:** cyanobacteria, mRNA decay, photosynthesis, termination, gene expression, machine learning, RNase, terminator, transcriptome

## Abstract

RNA degradation is an important process that influences the ultimate concentration of individual proteins inside cells. While the main enzymes that facilitate this process have been identified, global maps of RNA turnover are available for only a few species. Even in these cases, there are few sequence elements that are known to enhance or destabilize a native transcript; even fewer confer the same effect when added to a heterologous transcript. To address this knowledge gap, we assayed genome-wide RNA degradation in the cyanobacterium *Synechococcus* sp. strain PCC 7002 by collecting total RNA samples after stopping nascent transcription with rifampin. We quantified the abundance of each position in the transcriptome as a function of time using RNA-sequencing data and later analyzed the global mRNA decay map using machine learning principles. Half-lives, calculated on a per-ORF (open reading frame) basis, were extremely short, with a median half-life of only 0.97 min. Despite extremely rapid turnover of most mRNA, transcripts encoding proteins involved in photosynthesis were both highly expressed and highly stable. Upon inspection of these stable transcripts, we identified an enriched motif in the 3′ untranslated region (UTR) that had similarity to Rho-independent terminators. We built statistical models for half-life prediction and used them to systematically identify sequence motifs in both 5′ and 3′ UTRs that correlate with stabilized transcripts. We found that transcripts linked to a terminator containing a poly(U) tract had a longer half-life than both those without a poly(U) tract and those without a terminator.

**IMPORTANCE** RNA degradation is an important process that affects the final concentration of individual mRNAs, affecting protein expression and cellular physiology. Studies of how RNA is degraded increase our knowledge of this fundamental process as well as enable the creation of genetic tools to manipulate RNA stability. By studying global transcript turnover, we searched for sequence elements that correlated with transcript (in)stability and used these sequences to guide tool design. This study probes global RNA turnover in a cyanobacterium, *Synechococcus* sp. strain PCC 7002, that both has a unique array of RNases that facilitate RNA degradation and is an industrially relevant strain that could be used to convert CO_2_ and sunlight into useful products.

## Abstract (precis)

RNA degradation is an important process that affects the final concentration of individual mRNAs, affecting protein expression and cellular physiology. Studies of how RNA is degraded increase our knowledge of this fundamental process as well as enable the creation of genetic tools to manipulate RNA stability. By studying global transcript turnover, we searched for sequence elements that correlated with transcript (in)stability and used these sequences to guide tool design. This study probes global RNA turnover in a cyanobacterium, *Synechococcus* sp. strain PCC 7002, that both has a unique array of RNases that facilitate RNA degradation and is an industrially relevant strain that could be used to convert CO_2_ and sunlight into useful products.

## INTRODUCTION

RNA degradation is a fundamental process that can influence the amount of mRNA present in a cell. In bacteria, the abundance of a given RNA transcript, and ultimately any protein it encodes, is controlled by the balance of nascent transcription and RNA degradation. A suite of enzymes called RNases facilitate the bulk of RNA turnover. Although many RNases have been characterized, there is limited information on their precise *in vivo* sequence targets and how enzymes contribute to the turnover of individual mRNA. While general mechanistic models of global RNA turnover have been described and debated (1–3), tools capable of predicting the stability of a given RNA remain elusive. This is in part because the relationship between sequence elements, secondary structures, and other biophysical information on the rate of RNA decay remain poorly understood. The first step toward closing this knowledge gap is collecting global decay rates across the transcriptome of various species.

In prokaryotes, transcripts are quickly turned over, allowing for rapid response to environmental change via conditional transcription (4). Using antibiotics to stop transcription, global turnover rates have been quantified in many species via bulk RNA measurements, microarrays, and more recently RNA-sequencing data. Transcript half-lives are often on the order of minutes: Escherichia coli, 2.8 min, 4.7 min, and 6.8 min (5–7); Bacillus subtilis, 5 min (8); Bacillus cereus, 2.4 min (9); Lactococcus lactis, 5.8 min (10); Mycobacterium tuberculosis, 9.5 min (11); and Chlamydia trachomatis trachoma, 15 to 17 min (12). Short turnover times are logical for fast-growing species, but rapid turnover has also been observed in a slow-growing cyanobacterium, *Prochlorococcus* strain MED4, where the median half-life was 2.4 min despite doubling only once or twice per day (13).

In this study, we examined RNA degradation in another cyanobacterium, *Synechococcus* sp. strain PCC 7002 (PCC 7002), which is among the fastest-growing photoautotrophs (doubling time ∼2.5 h) (14). PCC 7002 is also genetically tractable with a wide suite of synthetic biology tools (15–18), making it attractive for fundamental studies and use for green chemical production from CO_2_ and sunlight (19–21). Another reason motivating the study of RNA degradation in PCC 7002 is its unique array of RNases that facilitate both RNA maturation and degradation. The functions of three homologs of RNase III have been studied (22), but PCC 7002 also has both an essential homolog of RNase E (a hallmark of RNA degradation in E. coli) and an essential homolog of RNase J (a hallmark of RNA degradation in B. subtilis) (23). The impact of the presence of both essential enzymes on RNA turnover remains unexplained. As a first step toward establishing a turnover model, we used RNA-sequencing to quantify global RNA levels in PCC 7002 after the arrest of transcription. We calculated global RNA half-lives on a per-ORF (open reading frame) basis and examined how transcript half-life was related to cellular function and what sequence features correlated with enhanced transcript stability. From this analysis, we observed that transcripts encoding proteins involved in photosynthesis were disproportionately stable, perhaps contributing to their large steady-state abundance. Using machine learning and motif identification algorithms, we identified a conserved sequence motif similar to Rho-independent terminators in the 3′ untranslated region (UTR) of these stable transcripts. These findings may guide the design of future heterologous transcripts and facilitate the development of global RNA turnover models.

## RESULTS

**FIG 1:**
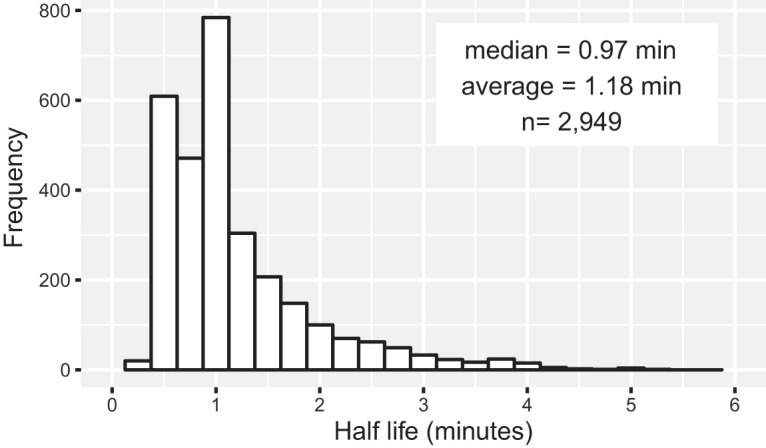
Global mRNA decay trends for PCC 7002. Distribution of half-lives for each transcribed ORF in PCC 7002. The median half-life of all analyzed genes (*n* = 2,949) was 0.97 min. The average half-life was 1.18 min (standard deviation = 0.73 min).

We collected total RNA samples from three biological replicates of PCC 7002 before (0 min) and 0.5, 1, 2.5, 5, 7.5, and 10 min after rifampin addition. We added synthetic RNA spike-ins to cell pellets immediately before lysis to normalize for potential large differences in mRNA pool sizes. We isolated total RNA and reduced the rRNA content with commercial reagents. We used this pool of enriched mRNA to prepare a library for sequencing via Illumina HiSeq. The number of reads that aligned to the RNA spike-ins was counted and found to linearly correlate with RNA copy number (see Fig. S1 in the supplemental material). After aligning each read to the PCC 7002 genome, the abundance of each position was normalized to the number of RNA spike-ins in the sample and averaged over each predicted ORF. We then fitted the normalized abundance to an exponential decay model and calculated the half-life of each ORF’s transcript. We were able to calculate transcript half-life values for 2,949 ORFs (91.1%) which had a median half-life of 0.97 min (Fig. 1). The average half-life was 1.18 min with a standard deviation of 0.73 min. We classified 0.5% of the transcripts as stable because each had greater than 50% of the original read counts in the final time point (*t* = 10 min). We could not calculate half-lives for 4.4% of transcripts because of null or low expression and for another 4.1% that exhibited a poor fit to the exponential model. For transcripts containing operons, we observed an increase in half-life along the length of the transcript (Fig. S2 and S3).

10.1128/mSystems.00224-20.1 FIG S1 Transcriptome sequencing (RNA-seq) read counts from RNA spike-ins. The read counts from RNA spike-ins from example replicate B time 0 minutes is shown. There is a linear relationship between the log read count of the spike-ins (normalized for length) and the log spike-in copy number for spike-ins added between 0.1 and 100 copies/cell. The spike-in added at 1,000 copies/cell was retrospectively too high and not used in the normalization calculation. The linear fit for the remaining 5 spike-ins is shown along with the shaded 95% confidence interval, *R*^2^ = 0.996. Download FIG S1, EPS file, 1.2 MB. Copyright © 2020 Gordon et al. 2020 Gordon et al. This content is distributed under the terms of the Creative Commons Attribution 4.0 International license.

10.1128/mSystems.00224-20.2 FIG S2 Half-life increases along transcript. The half-lives of genes from eight operons from PCC 7002 are shown with the points located in the middle of the gene. The bottom diagram shows the number of genes, their spacing, and locus tags. The approximate transcriptional start site is +1. Download FIG S2, EPS file, 1.4 MB. Copyright © 2020 Gordon et al. 2020 Gordon et al. This content is distributed under the terms of the Creative Commons Attribution 4.0 International license.

10.1128/mSystems.00224-20.3 FIG S3 Inferences gleaned from regression coefficients in the model built using support vector regression with linear kernels for predicting half-lives using 12 sequence- and structure-based features for 2,587 genes in PCC 7002. The model yielded a correlation coefficient of 0.398 between predicted values and actual values and a root mean square error (RMSE) of 0.69 min (∼13.33% of maximum half-life value in the data set and within the noise level observed in experimentally measured replicates). Download FIG S3, EPS file, 1.2 MB. Copyright © 2020 Gordon et al. 2020 Gordon et al. This content is distributed under the terms of the Creative Commons Attribution 4.0 International license.

**FIG 2:**
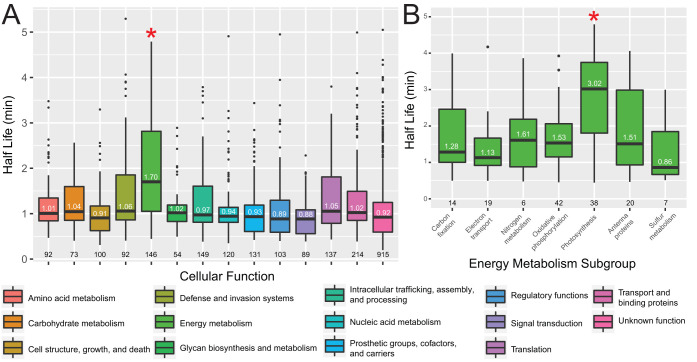
Photosynthesis genes have longer half-lives than genes classified with other cellular functions. (A) Half-lives of genes grouped by cellular function. Only cellular functions that contained greater than 50 genes were included. The median half-life is displayed on the boxplot, and the number of genes in each group is displayed below. The energy metabolism group had a significantly greater half-life than all other groups (*, pairwise *t* test *P* values between 9 × 10^−6^ and 2 × 10^−16^, Bonferroni adjusted). (B) Half-lives of genes in the energy metabolism subgroup. The photosynthesis group had a significantly greater half-life than all other groups (*, pairwise *t* test *P* values all below <0.05, Bonferroni adjusted).

We grouped the half-life of protein coding transcripts by cellular function and found that transcripts encoding proteins involved in energy metabolism had a longer half-life than all other groups (Fig. 2A, pairwise *t* test *P* values between 9 × 10^−6^ and 2 × 10^−16^, Bonferroni adjusted). Only cellular functions that contained greater than 50 genes were included in the analysis. Within the energy metabolism subgroup, transcripts created from the photosynthesis genes had a longer half-life than all other subgroups (Fig. 2B, pairwise *t* test *P* values all below <0.05, Bonferroni adjusted).

**FIG 3:**
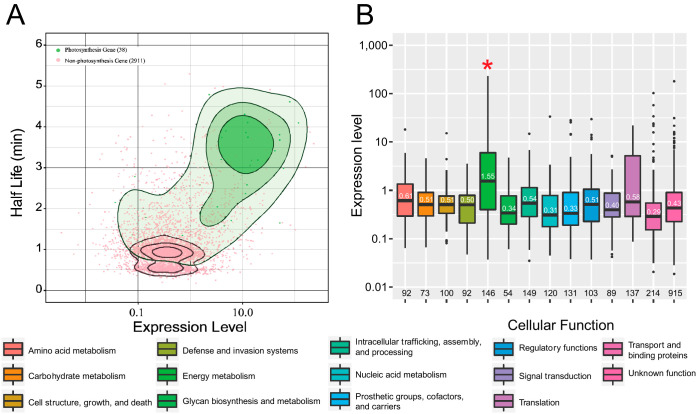
Photosynthesis genes are highly expressed and highly stable. (A) The half-life and expression level of photosynthesis genes (green) and nonphotosynthesis genes (pink) are shown along with contours of the two-dimensional kernel density estimation. (B) Energy metabolism genes have a higher expression level than genes with other cellular functions (*, pairwise *t* test *P* values between 2 × 10^−16^ and 3 × 10^−11^, Bonferroni adjusted). The median expression level is displayed on the boxplot, and the number of genes in each group is displayed below.

We looked for a relationship between transcript level and half-life and saw no overall trend (*r* = 0.13, Pearson correlation), but photosynthesis genes seemed to be highly transcribed and their transcripts seemed to be long-lived (Fig. 3A) (*r* = 0.40, Pearson correlation). Transcripts encoding proteins involved in energy metabolism were more abundant than transcripts encoding proteins involved with other cellular functions (Fig. 3B, pairwise *t* test *P* values between 2 × 10^−16^ and 3 × 10^−11^, Bonferroni adjusted), but photosynthesis transcripts were not more abundant than those within the energy metabolism subgroup (Fig. S4). Instead, only the antenna protein group had statistically significant higher expression than all other subgroups besides nitrogen metabolism.

10.1128/mSystems.00224-20.4 FIG S4 Expression level within energy metabolism subgroup. The antenna protein group had a significantly higher expression level than all other groups besides nitrogen metabolism (*, pairwise *t* test *P* values < 0.05, Bonferroni adjusted). Download FIG S4, EPS file, 1.3 MB. Copyright © 2020 Gordon et al. 2020 Gordon et al. This content is distributed under the terms of the Creative Commons Attribution 4.0 International license.

**FIG 4:**
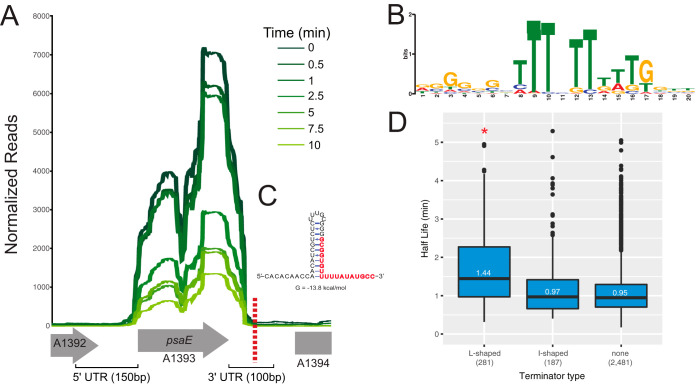
L-shaped Rho-independent terminators are enriched in stable genes. (A) RNA degradation of photosynthesis gene A1393 (*psaE*) throughout time. The number of normalized reads aligning to each base is shown in the samples taken from different time points following addition of rifampin (dark green to light green). (B) The motif enriched in the 100 bases downstream (3′ UTR) of the top 400 stable ORFs compared to the 400 least stable ORFs (MEME E value of 5.6e−083). (C) The terminator sequence of A1393 predicted by RNAMotif (19) that contains the enriched motif. (D) Genes with an L-shaped terminator in the 3′ UTR have an increased half-life compared to genes with an I-shaped terminator or those that lack a terminator (*, pairwise *t* test *P* values: L versus I, 4.8 × 10^−13^, and L versus none, <2 × 10^−16^, Bonferroni adjusted).

While examining the read coverage, we observed that highly stable transcripts had very distinct start and stop locations (Fig. 4A). We looked for motifs in both the upstream region (150 bp), an approximation of the 5′ UTR, and downstream region (100 bp), an approximation of the 3′ UTR, of each ORF that were enriched when linked to transcripts with long half-lives. Using MEME (24) in discriminative mode, we looked for motifs overrepresented in the sequences surrounding the 400 most stable transcripts compared to the control 400 least stable transcripts. No significant motifs were identified in the upstream region, but an enriched motif was found in the downstream region (Fig. 4B). This 21-base motif had an E value of 5.6e^−083^ and featured a prominent string of six consecutive U’s. This motif is reminiscent of Rho-independent terminators, which feature a G-C-rich hairpin followed by a U-rich tract. Therefore, we asked whether the presence of a terminator in the 3′ untranslated region (UTR) was predictive of stability of other transcripts. To examine whether different putative terminator forms are significantly associated with transcript stability, we used the Web Genome Scanner for Terminators (WebGeSTer) database (25). We divided transcripts into three classes based on the absence of a known terminator motif, presence of an L-shaped terminator (hairpin with a U-tract), or the presence of an I-shaped terminator (hairpin without a U-tract). We found that transcripts linked to an L-shaped terminator containing a U-tract in the 3′ UTR had a significantly longer half-life (*P = *4.8 × 10^−13^ [L versus I] and *P < *2.0 × 10^−16^ [L versus none]) than transcripts that contained an I-shaped terminator in the 3′ UTR or no obvious Rho-independent terminator (Fig. 4D). Additionally, bootstrap sampling was performed to assess the effect of various sample sizes between the subsets of genes with and without terminators (Table S1).

10.1128/mSystems.00224-20.7 TABLE S1 Median half-life values with and without bootstrap sampling to account for variability in sample size. Download Table S1, PDF file, 0.1 MB. Copyright © 2020 Gordon et al. 2020 Gordon et al. This content is distributed under the terms of the Creative Commons Attribution 4.0 International license.

**FIG 5:**
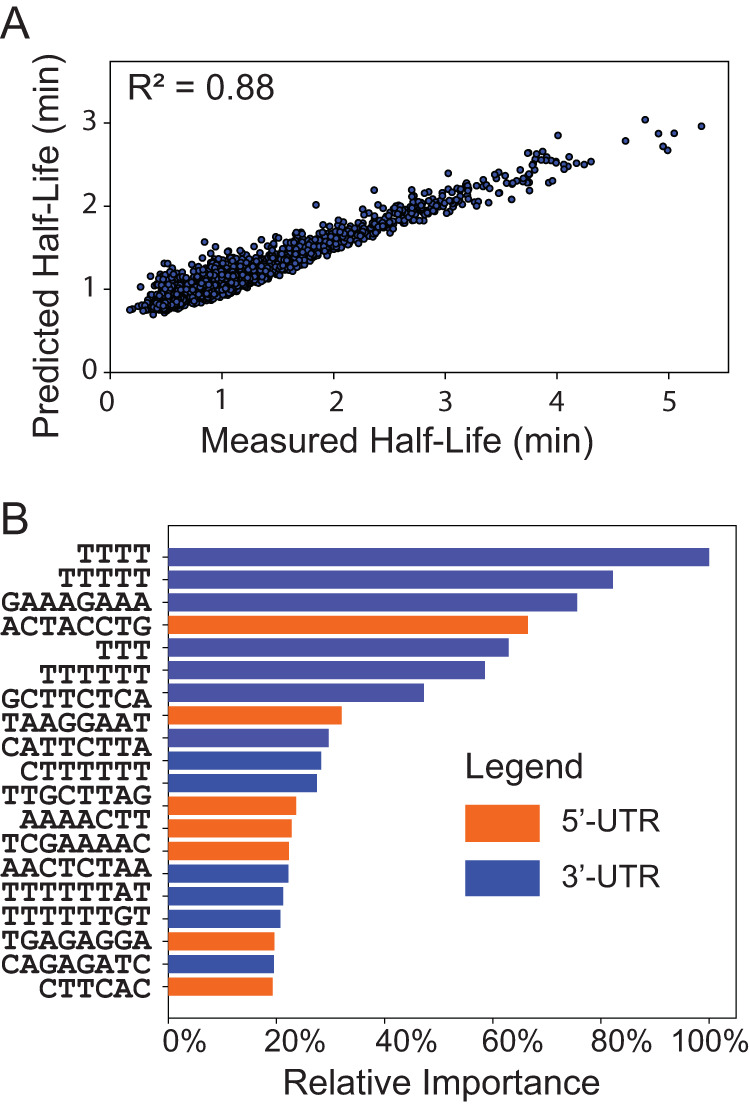
Machine learning-based identification of sequence motifs in UTR. (A) Scatterplot illustrating the predictive performance of the random forest regression model built using counts of 3- to 8-lettered sequence motifs as features. (B) Top 20 motifs across 5′ and 3′ UTRs based on feature importance scores.

In order to systematically analyze the effect of sequence-based elements, the counts of all possible 3- to 8-letter-long sequence motifs present in the 5′ and 3′ UTRs were used as features in a random forest model built to search for additional motifs. Model-predicted half-life values for different transcripts in PCC 7002 correlated very well with experimentally measured half-lives (Spearman rank coefficient of 0.88 under 10-fold cross validation [Fig. 5A]). The feature importance scores revealed a set of putative sequence motifs in the 5′ and 3′ UTRs that correlate with transcript stability and therefore could be used to enhance the stability of a heterologous transcript. Consistent with the prior MEME search, most of the motifs are present in the 3′ UTR and contain a poly(U) trail which could be involved in Rho-independent termination. A few motifs in the 5′ UTR were detected (e.g., ACTACCTG, TAAGGAAT, AAAACTT, TCGAAAAC, and AACTCTAA). In addition to enhancing the stability of mRNA transcripts, these sequence motifs might be involved in enhancing translation initiation (26). Although one of the motifs has a purine-rich signal (AGGA) that resembles the Shine-Dalgarno (SD) sequence, it is well known that SD-like sequences found in the leaders of many mRNAs from cyanobacteria are hypervariable in base composition, size, and location compared to those in E. coli (27).

## DISCUSSION

RNA turnover in PCC 7002 is extremely rapid with a median half-life of coding regions of 0.97 min (∼58 s), faster than any other report of bacterial mRNA decay. Only 15 transcripts were classified as stable (retaining at least 50% of the read counts from the initial pre-rifampin time point). Prior experiments using RNA sequencing to study RNA degradation (5, 9) have shown that mRNA half-lives are much shorter than original values determined by microarrays (6–8). Even so, RNA degradation occurred more rapidly in PCC 7002 than expected.

We examined if any groups of transcripts that encode different cellular functions had significantly longer half-lives and found both that photosynthesis genes were highly expressed and that their transcripts were highly stable. Moreover, the biophysical feature-based analysis indicated expression level to be one of the top predictors of mRNA half-life (see Fig. S3 in the supplemental material). The abundance of mRNA-encoding photosynthesis genes and all genes falling under the energy metabolism category is not by itself surprising, as these proteins perform essential functions in the cell. There are only 53 genes categorized as photosynthesis genes, of which the mRNA half-life values could be determined for 38 and 2 were categorized as stable. Many of these 40 transcripts are both highly abundant and very stable, making it hard to differentiate between whether being inherently stable increases expression level and/or having high expression protects the transcript from degradation.

Nevertheless, we wanted to identify sequence elements that may be predictive of increased transcript stability; to precisely control RNA transcript stability, these elements may be helpful to guide the construction of heterologous transcripts. To this end, we identified an enriched motif in the 3′ UTR of stable transcripts that consisted of a string of U’s. Upon further characterization, putative upstream hairpins were also identified, which suggests that this motif is reminiscent of canonical Rho-independent terminators that consist of a G-C-rich hairpin followed by a U-tract. Indeed, the location of these motifs had very significant overlap with computationally predicted Rho-independent terminators (28, 29). We then explored whether putative terminator forms may be associated with transcript stability. Early algorithms designed to identify intrinsic terminators searched for the canonical sequence motifs that are typically found in E. coli intrinsic termination: the GC palindrome and the poly(U) tract. Surprisingly, in many organisms no terminators were found, and it was suggested that they relied on some yet-uncharacterized termination mechanism (30). More recent algorithms designed to address this issue found that many of these organisms contain hairpin structures but lack the canonical U-tract and were thus overlooked (31). Terminators have been classified into several groups with the major one being L-shaped (hairpin and U-tract) and I-shaped (hairpin with no U-tract). We found that transcripts with L-shaped terminators had a significantly longer half-life than those with an I-shaped terminator or no predicted terminator in the 3′ UTR, which strongly suggests that terminator form is predictive of transcript stability in PCC 7002. That said, initial attempts to stabilize heterologous transcripts with sequences pulled from the stable cassettes failed.

The observation that an L-shaped terminator may be associated with stable transcripts in PCC 7002, but not an I-shaped terminator, indicates that the U-tract itself may play an important function in contributing to transcript stability. This could be a direct effect through the affinity and activity of exoribonucleases on a terminal U-tract or could be indirect, causing a difference in termination efficiency. It has been suggested that the U-tract serves as a pausing signal (possibly through backtracking) that facilitates the folding of the hairpin, irreversibly trapping the RNA polymerase complex and disrupting all key contacts between RNA, DNA, and RNA polymerase (32). It is possible that the presence or absence of the U-tract determines termination efficiency and the presence or absence of the poly-U’s at the end of a transcript influences transcript stability. The distinction between different Rho-independent termination structures could be quite important in cyanobacteria because without a known homolog of Rho (33, 34), intrinsic termination is likely the dominant termination method. However, the precise reason for this association with stable transcripts remains unknown and could be, likely, coupled to the specific RNA transcript sequences upstream or downstream of the putative L-form terminators. This is especially true as many transcripts with putative L-form terminators have comparable or lower stability than transcripts without this putative terminator form. Moreover, around one-fourth of the genes in the energy metabolism group, as well as the subcategory photosynthesis genes, have L-shaped terminators (Fig. S5), indicating the role of a gene and the presence of an L-shaped terminator to be jointly affecting the stability of a gene.

10.1128/mSystems.00224-20.5 FIG S5 Role of a gene and occurrence of L-shaped terminators. (A) The category of “energy metabolism” genes has the maximum number of genes with L-shaped terminators (upon excluding the genes with unknown function). (B) Distribution of terminator type among 146 energy metabolism genes. (C) Distribution of terminator type among 38 photosynthesis genes (a subcategory of energy metabolism). Download FIG S5, EPS file, 0.1 MB. Copyright © 2020 Gordon et al. 2020 Gordon et al. This content is distributed under the terms of the Creative Commons Attribution 4.0 International license.

The possibility that the presence and characteristics of a terminator may enhance stability of a transcript is not unprecedented. The impact of both the hairpin and U-tract on transcript stability of the E. coli *crp* transcript was analyzed, and the removal of the of the G-C hairpin enhanced transcript degradation, but disruption of the U-tract did not affect transcript stability. Additionally, swapping the terminator for other native Rho-independent terminators did not change expression level or transcript stability (35). However, as E. coli was the model organism tested in this study, it is reasonable to speculate that RNAs may be processed differently in cyanobacteria, especially given a different repertoire of RNases (23). Indeed, the presence of RNA hairpins at the 3′ end of transcripts may act as a barrier to exonuclease degradation, as several 3′-to-5′ exonucleases cannot degrade secondary structure (RNase II) or require a toehold such as polyadenylation to initiate degradation (RNase R and PNPase) (36). Nevertheless, it remains to be seen whether RNases act differently on L- or I-form terminators and, thus, influence transcript stability in a noncanonical 3′-to-5′ direction.

Machine learning-based statistical models were built to systematically explore the role of different sequence- and structure-based properties of mRNA in the stability of a transcript. Analyzing the regression coefficients in this model revealed that the normalized expression level of a transcript, its position within the operon, and the ribosome binding site (RBS) strength as predicted by the RBS calculator (37) were three of the most influential factors (Fig. S3). The effects of expression level and position within an operon have already been discussed in the preceding paragraphs. A plausible reason for observing RBS strength as one of the top three influential factors is that the genes that are being actively translated might be less susceptible to the nuclease activity.

Several aspects of this data collection and workflow were fundamental to obtaining high-quality half-lives for most of the PCC 7002 transcriptome. The short time frame of sampling was extremely important for capturing rapidly decaying messages. Most studies of RNA degradation have taken an initial time point and then not collected the next samples until 2.5 or 5 min after addition of rifampin. In all of these studies, the overall median or average half-life is likely skewed as much of the early mRNA degradation may have been missed. A study in E. coli that included an 0.5-min time point also found an extremely low median half-life of 2.8 min in exponential phase and 5.4 min in stationary phase (5).

Another critical aspect of this data set is the use of RNA spike-ins (5). The typical RNA-sequencing pipeline normalizes to the total number of reads from each sample, which can greatly distort the results when the pool size of mRNA is shrinking throughout the time course. RNA spike-ins can be added proportionally to the number of cells to normalize both for the change in RNA pool size between samples and for variation between samples introduced during library preparation. The need for RNA spike-ins to correct for differences in RNA or DNA yields from samples has been addressed previously (38), but their use has still been quite limited. A standardized set of spike-ins has been developed for eukaryotes (the External RNA Controls Consortium [ERCC] mix of 96 polyadenylated transcripts), but there is no standardized set for use in prokaryotes. We hope that RNA spike-ins become standard for future RNA degradation studies and that the methods described in this work and reference 5 will enable robust measurements of RNA half-lives in other prokaryotes.

This data set can also be probed to ask other fundamental questions about RNA degradation such as the potential directionality of transcript degradation. Early E. coli measurements using microarrays suggested that degradation occurs in a 5′-to-3′ direction by analyzing operons containing 2 or more ORFs (7). Similar findings were found in the slow-growing cyanobacterium *Prochlorococcus* MED4, where stability correlated with distance from the transcription start site (13). This 5′-to-3′ directionality was also observed within monocistronic transcripts. In B. cereus, researchers observed a mix of 5′-to-3′ degradation, 3′-to-5′ degradation, and more complex patterns (9). Here, we also observed a 5′-to-3′ directionality of degradation where the half-life increased with distance along an operon (Fig. S2 and S3). The 5′-to-3′ directionality may be caused by strong RNase J (5′-to-3′ exonuclease) activity or RNase E processively cleaving transcripts in this direction.

Alternatively, the observed 5′-to-3′ directionality may be caused by the use of rifampin to stop transcription. Rifampin binds the β subunit of RNA polymerase and blocks the channel for the elongating RNA (39). This blockage prevents extension of the RNA beyond 2 or 3 nucleotides but has no effect on elongating RNA polymerases. Researchers have noticed a delay before transcripts begin to decay for locations downstream of the transcription start site (5). They showed that this delay disappeared when streptolydigin (an antibiotic that prevents transcription elongation) was used in place of rifampin. The change in this delay due to residual transcription was used to calculate the elongation rate, 25 nucleotides per second on average. The effect of residual transcription after rifampin addition was estimated to be less than 30 s for E. coli (40).

Despite these drawbacks, rifampin has been used in all RNA-sequencing and microarray studies to examine global transcript degradation. This is due to rifampin availability and cost. The confounding influence on half-life calculations may be minimal when initial time points are not taken until 2.5 or 5 min after rifampin addition, but it may significantly influence half-lives when samples are taken early. This effect would be even more exaggerated with greater global transcription elongation rates. There is little known about cyanobacterial transcription rates, but *in vitro* data comparing RNA polymerase from *Synechococcus* sp. strain PCC 7942 and Thermosynechococcus elongatus BP-1 with that from E. coli showed that the cyanobacterial enzymes took six times longer to complete the same transcript (41). Additionally, there were significant differences in levels of abortive transcription and misincorporation. The global differences in transcriptional machinery and transcription rates must also be considered when comparing global degradation rates between organisms. We hypothesize that the 5′-to-3′ directionality we see in degradation in this cyanobacterial data set may be entirely due to the use of rifampin and residual transcription.

The conditions during sampling may have a large impact on RNA half-life. These cyanobacterial RNA half-lives were determined at an optical density (OD_730_) of ∼0.2, which is between late exponential and early linear phase for the environmental conditions used. We chose this growth phase because we wanted to examine actively growing and dividing cells. This could be why we saw high stability and abundance of mRNA encoded by photosynthesis genes. In this time period, cells were growing rapidly and needed to produce high levels of photosynthesis machinery. Transcript degradation, however, is likely influenced by many factors including growth phase, growth rate, and environmental changes. There is an increasing body of evidence that RNase activity and expression can be influenced by inhibitors (42), temperature (43), growth phase (44), and osmolarity (45). The findings described here are from one experimental condition and would likely be different under other conditions. To gain a better understanding of global RNA degradation, it will be necessary to examine global decay rates under many different conditions and to identify which specific RNases are affected under these conditions. Ultimately, a global understanding of transcript degradation would enable accurate predictions of transcript half-life given its sequence, but currently there is a lack of knowledge of how transcript stability is regulated and how different sequence elements impact RNA processing and degradation.

## MATERIALS AND METHODS

### Sampling.

Wild-type *Synechococcus* sp. PCC 7002 (Pasteur Culture Collection) was cultivated in a temperature-controlled environment (37°C) with light provided by cool white fluorescent lights (4,100 K). A −80°C frozen stock of PCC 7002 was streaked on A+ medium (46) solidified with 1.5% agar and grown under continuous illumination at 115 μmol photons · m^−2^ · s^−2^ for 4 days and then used to inoculate liquid cultures. All liquid cultures were bubbled with air and continuous illumination of 215 μmol photons · m^−2^ · s^−2^. A glass culture tube containing 21 ml A+ medium was inoculated from plates and grown for 24 h. The OD_730_ was measured and used to inoculate 3 separate bubble tubes containing 21 ml A+ medium at an OD_730_ of 0.1, which were grown for 24 h. The OD_730_ was measured, and 3 separate 1-liter bottles containing 900 ml of A+ medium were inoculated with preculture to achieve an OD_730_ of 0.01. Cultures were bubbled with air and grown with continuous illumination for 22 h to reach an OD_730_ of ∼0.2 before sampling.

Two milliliters of each culture was collected for cell counts via hemocytometer and OD_730_ measurements. Forty milliliters of culture was collected, deposited into a 50-ml conical tube containing 5.0 ml stop solution (10% phenol in ethanol), and placed on ice. Rifampin was added at a concentration of 200 μg/ml (in dimethyl sulfoxide [DMSO]), and samples were taken as described above at 0.5, 1, 2.5, 5, 7.5, and 10 min following addition. Samples were spun down in a Beckman Coulter Avanti J-E centrifuge at 7,500 × *g* for 10 min at 4°C. The supernatant was carefully aspirated to prevent loss of cells, and samples were flash frozen in liquid nitrogen and stored at −80°C until RNA extraction (∼1 week).

Processes regulated by circadian rhythms or light-dark cycles could have significant and important influences on gene expression levels and decay rates in addition to overall cellular physiology. To avoid complexity introduced by these variables, continuous illumination was used for precultures and experimental growth conditions. Several tradeoffs were considered during experimental design. First, because light shading in optically dense cultures attenuates growth, we chose to use rapidly growing dilute cultures at late exponential/early linear growth phase (OD_730_ of ∼0.2). Thus, dilute cultures were harvested via centrifugation. There was some flotation of the cultures which led to pelleting on the side of the vial, but since they were centrifuged in 50-ml vials, the supernatant could be carefully aspirated without disruption or loss of the pellet. Careful harvesting and extraction are crucial aspects of these experiments, and extra care was taken at each step. It is possible that residual RNase activity could affect the measurements. Therefore, we used a quench solution and rapid cooling to prevent residual activity, which would be much more likely before hot-phenol extraction. In addition, we added RNA spike-ins at the extraction step to account for residual RNase activity. The decay curves were fitted to an exponential decay model, and multiple replicates were used to compare samples and provide statistical support to the overall findings. Biological replicates can reduce the error due to potential RNase activity.

### RNA spike-in preparation.

RNA spike-ins were used for normalization to ensure that read counts corresponded to RNA copy number. Six RNA spike-ins from bacteriophage ϕX174 were used as in reference 5. DNA templates for *in vitro* transcription were created by including the T7 promoter sequence in the primer sequence (see Table S2 in the supplemental material) and amplifying ϕX174 DNA (New England Biolabs; catalog no. N3023S). DNA templates were purified with ethanol precipitation and used to synthesize RNA with the T7 RiboMAX Express large-scale RNA production system (Promega; catalog no. P1320). RNA was purified with ethanol precipitation and quantified via NanoDrop. RNA spike-ins were combined to obtain a range of copy numbers per cell (gene H, 0.1 copy/cell; gene D, 1 copy/cell; genes F and G, 10 copies/cell; fragment 290, 100 copies/cell; and fragment 190, 1,000 copies/cell).

10.1128/mSystems.00224-20.8 TABLE S2 Primers used to generate DNA templates for *in vitro* transcription of RNA spike-ins. Download Table S2, XLSX file, 0.01 MB. Copyright © 2020 Gordon et al. 2020 Gordon et al. This content is distributed under the terms of the Creative Commons Attribution 4.0 International license.

### RNA extraction and sequencing.

RNA spike-ins were added to samples immediately before extraction with hot phenol and DNase treatment as in reference 47. RNA was quantified via NanoDrop, quality verified with the Bioanalyzer, and submitted to the University of Wisconsin Madison Biotechnology Gene Expression Center for library preparation and sequencing. rRNA was removed with a Ribo-Zero magnetic kit, and cDNA was creating with a TruSeq stranded total RNA library kit. Libraries were sequenced on an Illumina HiSeq 2500 (1 × 100).

### RNA spike-in normalization and half-life determination.

Sequencing files were aligned to the PCC 7002 chromosome and plasmids (NC_010474 to NC_010480) as well as the ϕX174 genome (NC_001422) with Bowtie 2 (v. 2.2.6) and SAMtools (v 1.2). The HTSeq count function (v 0.6.1) was used to count the number of reads that aligned to each feature. A linear relationship between log RNA spike-in copy number and read counts was observed only for spike-ins added at a ratio of 0.01 to 10 copies/cell, so only these were used for normalization (Fig. S1). All PCC 7002 transcripts were within this range, and all samples had a good correlation between log RNA spike-in copy number and read count with all *R*^2^ values being between 0.971 and 0.999. The sum of the reads that aligned to the spike-ins was calculated for each sample and used to create a normalization factor to keep the sum of reads that aligned to the spike-ins constant throughout the time course.

We combined data from all 3 biological replicates and used all 21 points to fit an exponential decay model. Degradation of many transcripts was extremely rapid, and read counts were close to zero for many later time points, so we determined the best number of time points to include based on the fit. We used the half-life corresponding to the highest *R*^2^ fit of using the first 3, 4, 5, 6, or all 7 time points. Based on our ability to calculate a half-life, we classified all transcripts as either “decay,” “stable,” “low_r2,” or “no_reads.” A full list of transcripts and calculated half-lives can be found in Table S3 organized by ORF.

10.1128/mSystems.00224-20.9 TABLE S3 Half-lives of PCC 7002 transcripts. Download Table S3, XLSX file, 0.1 MB. Copyright © 2020 Gordon et al. 2020 Gordon et al. This content is distributed under the terms of the Creative Commons Attribution 4.0 International license.

### Motif enrichment.

Using MEME (24), we searched for enriched motifs in both the upstream 150-bp region and downstream 100-bp region of each ORF. Using discriminative mode, we looked for motifs enriched in the sequences of the 400 most stable transcripts compared to the control 400 least stable transcripts. We looked for site distributions of zero or one occurrence per sequence (zoops) and looked in the coding strand only. Top hits always included the highly iterative palindrome 1 (HIP1, 5′-GGCGATCGCC-3′) sequence. This sequence is a target of methylation in other cyanobacteria and may be involved in modes of DNA repair (48, 49). However, its role in PCC 7002 remains unexplained.

### Terminator analysis.

The types and locations of terminators were extracted from the Web Genome Scanner for Terminators Database (WebGeSTer DB) (25). The input parameters for identification of terminators were maximum stem length of 12, minimum stem length of 4, maximum loop length of 8, minimum loop length of 3, maximum mismatch of 3, maximum distance from ORF of 270, and a species-specific Δ*G* cutoff of −11.618 kcal/mol.

### Machine learning-based model building and analysis.

A variant of random forest regression, often referred to as extremely randomized trees-based regression (50), was used in this work. Conventionally, a random forest approach builds an ensemble model by combining decision trees built on bootstrap sampled data sets as well as using a random subset of features to identify the best candidate feature for splitting at each node (51). This randomization helps increase the variability among the individual decision trees built and also helps speed up the process of model building as one does not have to analyze the complete set of features (∼200,000 features for UTR sequence motif analysis). The extremely randomized trees approach uses an additional layer of randomization by randomly choosing thresholds at each node rather than computing the most discriminative threshold at each step. This helps reduce the variance component of error for the ensemble model built by averaging the predictions across individual trees at the cost of a minor increase in bias.

The optimal values for hyperparameters, max_features (number of features to consider when looking for best split expressed in terms of fraction) and min_samples_split (the minimum number of samples required to split an internal node), were found to be 0.5 and 100 based on a grid search over the range [0.1, 0.2, 0.3, 0.5, 0.7, 0.9] and [2, 5, 10, 50, 100, 200, 500, 1,000], respectively, under 10-fold cross-validation. Number of estimators was set to 100 decision trees.

The feature importance scores were computed based on mean decrease in impurity measures, and the distribution of sequence motif in training set was used to determine whether a particular motif is stabilizing or destabilizing in nature. In general, the features that show up at the top of a decision tree are considered more important as they contribute to predictive decision of a larger fraction of samples. The importance of a node in a decision tree can be determined by computing the decrease in impurity metric for the node weighted by the fraction of samples arriving at that node (Fig. S6). Feature importance scores can then be computed by averaging the node importance scores pertaining to the feature and then averaging it across all trees in the forest. For regression setting, the impurity metric is computed based on decrease in mean squared error (or variance), while for classification setting, the impurity metric is computed based on the Gini index which is defined as follows:

$${\text{Gini index}}_{j}=\overset{C}{\underset{i=1}{\sum }}f_{i}(1-f_{i})$$

where *j* is the node index, *i* is the label index, *c* denotes the cardinality of classes, and *f* is the fraction of samples belonging to class *i*.

10.1128/mSystems.00224-20.6 FIG S6 Feature importance computation using impurity measures. (A) Toy data set consisting of 5 samples. (B) Schematic decision tree. (C) Feature importance score under regression and classification settings. Download FIG S6, EPS file, 0.1 MB. Copyright © 2020 Gordon et al. 2020 Gordon et al. This content is distributed under the terms of the Creative Commons Attribution 4.0 International license.

### Biophysical feature-based maximum likelihood (ML) statistical analysis.

A set of 19 biophysical features ranging from simple sequence-based features such as length of the transcript and GC content of the coding region to mRNA secondary structure-based minimum free energy for folding were computed for each of the 2,949 genes in the half-life data set (Table S3). Binary features were converted into Boolean values (0 or 1), categorical features were converted into binary strings using one-hot encoding, and numerical features were normalized using min-max scaling. Later, support vector regression with linear kernel was used to predict half-life values and the values of regression coefficients were used to assess the relative importance of features.

### Accession number(s).

Raw sequencing files have been deposited in NCBI’s Sequence Read Archive (accession number SRP130967). Half-lives of transcripts and per-base-count data have been deposited at the Gene Expression Omnibus (accession number GSE109174).
