# Supplementary material for: Genome-Wide Analysis of RNA Decay in the Cyanobacterium Synechococcus sp. Strain PCC 7002
Source: mSystems. 2020 Aug 4;5(4):e00224-20. doi: 10.1128/mSystems.00224-20 (PMC7406221; doi:10.1128/mSystems.00224-20)
Supplement: TABLE S1 [file mSystems.00224-20-st001.pdf]

Median half-life values with and without bootstrap sampling to account for sample size

Bootstrap Sampling	L-shaped (n = 281)	I-shaped (n = 187)	None (n = 2,481)
Yes	1.46 min	0.97 min	0.95 min
No	1.44 min	0.97 min	0.95 min
